# Evaluation of cardiopulmonary and inflammatory markers in dogs with heartworm infection during treatment with the 2014 American Heartworm Society recommended treatment protocol

**DOI:** 10.1186/s13071-017-2427-7

**Published:** 2017-11-09

**Authors:** Won-Kyoung Yoon, Ye-Won Kim, Sang-I L Suh, Ran Choi, Seung-Gon Lee, Changbaig Hyun

**Affiliations:** Section of Small Animal Internal Medicine, College of Veterinary Medicine, Kangwon National University, 1 Kangwondaehak-gil, Chuncheon-si, Gangwon-do 24341 Republic of Korea

**Keywords:** AHS guidelines, Biomarker, Heartworm, Melarsomine, Interleukin 6

## Abstract

**Background:**

Heartworm disease in dogs is a life-threatening parasitic disease. Although adulticide treatment with melarsomine has been proven to be the most effective, complications associated with adulticide treatment are major concerns for clinicians.

**Methods:**

This study evaluated the change in levels of D-dimer, interleukin-6, C-reactive protein and cardiac troponin I in 12 dogs with different severities of heartworm infection treated by the American Heartworm Society (AHS) recommended protocol during the treatment period. The serum levels of several markers were measured on the day of diagnosis (T-60), before the initiation of melarsomine therapy (T0), 1 day after the first injection (T1), 1 week after the first injection (T7), 1 month after the first injection (T30), 1 day after the second injection (T31), 1 day after the third injection (T32), 1 week after the third injection (T39), 1 month after the third injection (T62), 2 months after the third injection (T92), 3 months after the third injection (T122), and 6 months after the third injection (T182).

**Results:**

The serum levels of these markers were significantly different at the test time point after melarsomine treatment and also differed significantly according to the stage of heartworm disease in the dogs.

**Conclusion:**

This study found that monitoring of inflammatory and hemostatic markers in dogs with heartworm disease being treated with melarsomine might be beneficial in predicting the clinical outcomes and complications associated with melarsomine treatment.

## Background

Heartworm disease (HWD) in dogs is a life-threatening parasitic disease caused by *Dirofilaria immitis* and is characterized by right-sided congestive heart failure, allergic pneumonia and thromboembolism [1]. Although several therapeutic modalities have been proposed [2–7], the 2014 American Heartworm Society (AHS) guidelines constitute the most popular and proven therapeutic protocol for dogs with HWD. This protocol includes three doses of melarsomine dihydrochloride along with a preventive dose of macrocyclic lactone and 4 weeks of doxycycline premedication [7]. Clinical outcomes and complications from this therapy differ by worm burden, host immune reaction to the clearance of dead worms, duration of infection and ability to restrict exercise. Therefore, monitoring myocardial damage, thromboembolism and inflammatory processes during the therapeutic period may play a key role in the prognosis and success of adulticide treatment.

A biomarker is a biological indicator reflecting local and systemic disease processes and/or response to a specific treatment [8]. Several types of biomarkers have been developed and widely used for screening and monitoring disease processes in human and veterinary fields [9–11]. Pathological processes in dogs with HWD during adulticide therapy may include myocardial injury and pulmonary thromboembolic and proinflammatory processes. Therefore, monitoring these processes using relevant biomarkers can be beneficial in increasing the therapeutic success and reducing the risk of complications.

Cardiac troponin I (cTnI) is a cardiac biomarker reflecting myocardial injury and has been proven to detect myocardial damage from cancer chemotherapy or certain heart diseases, including HWD [12, 13]. D-dimer is a marker for detecting thromboembolic processes and has been found to be sensitive in detecting dogs with high risks of thromboembolism, including immune-medicated hemolytic anemia, disseminated intravascular coagulation and HWD [14–17].

C-reactive protein (CRP) is an acute phase protein reflecting the inflammatory processes in response to infection or trauma and has been well correlated with the severity of inflammatory and degenerative diseases in dogs [18]. Interleukin-6 (IL-6) is also an acute phase protein involved in inflammatory processes, including septicemia and the inflammatory response syndrome (SIRS) [19], but has never been studied in dogs with HWD. The objective of this study was to evaluate the change in cardiopulmonary and inflammatory biomarkers (ie, cTnI, D-dimer, CRP and IL-6) during adulticide therapy in dogs with different severities of HWD.

## Methods

### Study population

Twelve dogs infected with heartworms were enrolled in this study. All dogs were brought from a private animal shelter. Consent was obtained from the owner of the private animal shelter for the participation of the dogs in this study. Heartworm infection in this study population was diagnosed using a commercial ELISA test kit (SNAP® 4Dx® Plus Test, IDEXX Laboratories, USA), as instructed by the manufacturer’s manual. The presence of microfilariae was further evaluated by a modified Knott’s test. Dogs were divided into three groups (*n* = 4 per group) based on the severity of the disease described in the 2014 AHS guidelines. As adulticide therapy is not recommended for dogs with Class IV (caval syndrome), they were not included in this study. The severity of HWD in each dog was confirmed by physical, radiographic and echocardiographic examinations. In brief, dogs included in class I have no clinical signs and are without abnormal findings in physical, radiographic or echocardiographic examinations, whereas in class II, mild clinical signs (ie, occasional cough with or without exercise intolerance) are present with abnormal findings in radiographic (ie, mild pulmonary infiltration and pulmonary arterial dilation) and echocardiographic examinations (ie, dilation of the main pulmonary artery without interventricular septal flattening).

**Fig. 1:**
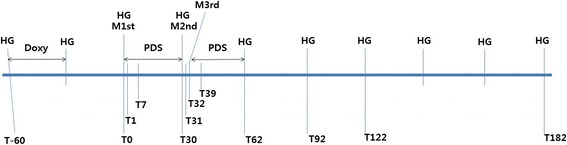
Medication and sampling protocol used in this study. HG: day of preventive dose of ivermectin administration; Doxy: day and period of doxycycline administration; M1st, M2nd, M3rd: day of melarsomine injection; T = study day

In class III, dogs have persistent and severe clinical signs (ie, persistent coughing, severe exercise intolerance, weight loss, cachexia and signs related to right-sided congestive heart failure) on radiographic (ie, marked pulmonary infiltration with pulmonary arterial dilation and right ventricular enlargement) and echocardiographic examinations (ie, dilation of the main pulmonary artery, tricuspid regurgitation [>2.8 m/s of peak velocity] along with pulmonary hypertension [>2.2 m/s of peak velocity]). The therapeutic protocol in this study strictly followed the 2014 AHS guidelines, except with regard to steroid therapy. Medication and sampling time points are illustrated in Fig. 1 **.**

### Laboratory evaluations

Blood samples were drawn from the cephalic vein of each animal for measurement of the concentrations of the biomarkers and were immediately centrifuged at 4000×*g* for 10 min. Three aliquots of plasma were immediately stored either at −80 °C in a freezer until testing (IL-6) or in dry ice until transportation to the reference laboratory (cTnI, D-dimer and CRP). The cTnI, D-dimer and CRP values in this study population were determined by reference laboratories (cTnI by IDEXX Laboratories, Sungnam, Korea; D-dimer and CRP by Neodin Vetlab, Seoul, Korea). Reference intervals of cTnI, D-dimer, CRP and IL-6 for healthy dogs were established as 0–0.08 ng/mL, 0–0.25 μg/mL, 0–17 mg/mL and 2–54 pg/mL, respectively, according to the reference laboratories. Canine IL-6 enzyme-linked immunosorbent assay (ELISA) was purchased and used within a week of receiving the kits (USNNK, Houston, Texas 77,084, USA).

The interassay and intraassay coefficients of variation for the assay were 5.6% and 7.9%, respectively, with the lowest and highest limits of detection being 5.0 and 250 pg/mL, respectively. The serum levels of each marker were measured on the day of diagnosis (T-60), before the initiation of melarsomine therapy (T0), one day after the first injection (T1), one week after the first injection (T7), one month after the first injection (T30), one day after the second injection (T31), one day after the third injection (T32), one week after the third injection (T39), one month after the third injection (T62), two months after the third injection (T92), three months after the third injection (T122), and six months after the third injection (T182; Fig. 1).

### Statistical analyses

Statistical analyses were performed using statistical software (MedCalc 12.1.3.0 for Windows, Belgium) throughout this study. Descriptive statistics were generated, and normality testing with the Kolmogorov–Smirnov test for all continuous variables was performed. Data are reported as median and range unless otherwise stated. Differences in continuous data among different HWD class groups were determined by Kruskal–Wallis tests. Medians of each biomarker at baseline (T-60) were compared to each time point after the initiation of the therapy using Mann–Whitney U tests. A value of *P* < 0.05 was considered statistically significant.

## Results

**Fig. 2:**
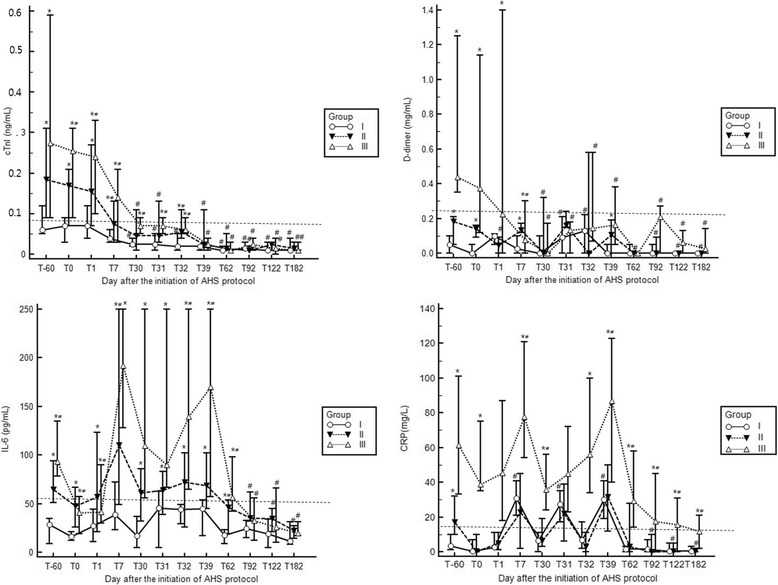
Changes in levels (median and range) of each biomarker at each time point before and after the adulticide therapy in heartworm-infected dogs treated according to 2014 AHS guidelines. See Fig. 1 for time point (T) indicated in this figure

The ages of the dogs enrolled in this study could not be determined, although all dogs were mature. The body weights of the dogs in class I, II and III groups were 6.8 ± 1.3 kg (one male and three females), 8.1 ± 3.0 kg (two males and two females), and 7.7 ± 4.2 kg (two males and two females), respectively. All dogs survived, and no dogs tested positive for the HWD antigen and microfilaria tests at the end of the study (T182). There was significant difference in all biomarker levels among the study groups at the first presentation (*P* < 0.05; Table 1 and Fig. 2). Most biomarker levels were significantly altered from the baseline (T-60) during the therapeutic period (see Table 1 and Fig. 2).

**Table 1:** Concentrations of serum biomarkers evaluated throughout the adulticide therapy in heartworm-infected dogs treated according to 2014 AHS guidelines. See Fig. 1 for time point (T) indicated in this table

Markers		T-60	T0	T1	T7	T30	T31	T32	T39	T62	T92	T122	T182
cTnI	I (median)	.06	.07	.07	.04	.03^#^	.03^#^	.02^#^	.02^#^	.01^#^	.02^#^	.01^#^	.01^#^
	Min	.05	.03	.04	.03	.02	.01	.01	.01	.01	.01	.01	.01
	Max	.12	.09	.12	.06	.04	.05	.05	.02	.02	.03	.03	.04
	II (median)	.19^*^	.17^*^	.16^*^	.08^*,#^	.05^#^	.05^#^	.06^*,#^	.03^#^	.02^#^	.01^#^	.03^#^	.02^#^
	Min	.09	.07	.07	.03	.01	.03	.04	.01	.01	.01	.01	.01
	Max	.31	.21	.27	.13	.11	.13	.11	.11	.05	.02	.03	.03
	III (median)	.28^*^	.26^*^	.24^*#^	.14^*#^	.07^*,#^	.07^*#^	.06^*#^	.03^#^	.01^#^	.03^#^	.02^#^	.01^#^
	Min	.09	.09	1.0	.03	.05	.05	.05	.01	.01	.01	.00	.01
	Max	.59	.31	.33	.21	.09	.09	.09	.04	.03	.04	.04	.03
D-dimer	I (median)	.05	.00	.10	.03	.00	.09^#^	.13^#^	.00	.00	.00	.00	.00
	Min	.00	.00	.05	.00	.00	.00	.00	.00	.00	.00	.00	.00
	Max	.10	.05	.11	.10	.10	.21	.22	.05	.05	.05	.00	.00
	II (median)	.19^*^	.14^*^	.05^#^	.14^*^	.08^#^	.16	.00	.11^*^	.00^#^	.00^#^	.00^#^	.00^#^
	Min	.05	.09	.00	.12	.00	.15	.00	.00	.00	.00	.00	.00
	Max	.21	.16	.09	.17	.32	.24	.58	.19	.00	.09	.00	.00
	III (median)	.44^*^	.38^*^	.23^*^	.08^*#^	.00^#^	.14^#^	.15^#^	.17^#^	.00^#^	.21^#^	.06^#^	.03^#^
	Min	.35	.12	.00	.00	.00	.00	.07	.05	.00	.00	.00	.00
	Max	1.25	1.14	1.40	.30	.17	.18	.58	.38	.00	.27	.13	.14
CRP	I (median)	4	1	3	31^#^	7	28^#^	7	30^#^	2	2	1	1
	Min	1	0	2	21	0	17	2	19	0	0	0	0
	Max	10	7	11	41	11	35	17	41	3	7	5	3
	II (median)	17^*^	1	5	23	7	22	3	32	3	0^#^	1^#^	1^#^
	Min	10	0	1	2	3	6	0	12	0	0	0	0
	Max	32	10	11	45	19	39	11	50	28	10	5	2
	III (median)	62^*^	39^*^	46^*^	78^*#^	36^*#^	45	56^*^	87^*#^	30^*#^	18^*#^	16^*#^	12^*#^
	Min	33	35	18	54	24	23	34	40	14	0	0	2
	Max	101	75	87	121	56	72	100	123	58	45	31	21
IL-6	I (median)	28	17	28	39	17	46	44	45	18	25	19	12
	Min	9	12	11	23	5	5	29	17	9	15	5	8
	Max	35	21	44	72	37	63	49	54	23	33	34	31
	II (median)	65^*^	48^*^	57^*^	111^*#^	61^*^	64^*^	72^*^	69^*^	47^*#^	35^#^	35^#^	23^#^
	Min	51	26	21	49	32	39	26	45	19	32	21	14
	Max	94	67	123	250	86	83	102	102	54	62	45	27
	III (median)	93^*^	41^*#^	41^*#^	192^*^	110^*^	90^*^	139^*#^	170^*#^	57^*#^	33^#^	26^#^	20^#^
	Min	78	19	30	128	56	67	65	57	42	12	10	17
	Max	135	57	90	250	250	250	250	250	98	56	66	31

Abbreviations: *cTnI* cardiac troponin I, *CRP* C-reactive protein, *IL-6* interleukin 6

*P < 0.05 class I to class II and III

^*#*^ *P* < 0.05 baseline value (T-60) to each time point

The cTnI levels at the first presentation (T-60) were significantly higher in dogs with severer clinical signs (*P* < 0.05; see Table 1 and Fig. 2). A pathological level of cTnI was found in 25% in class I and 100% in class II and III groups, although no dog in any group had a pathological level of cTnI at the end of the study (T182). The cTnI levels in all of the classes of dogs decreased significantly after initiation of the therapy (*P* < 0.05; see Table 1 and Fig. 2). The cTnI levels did not change significantly after melarsomine injection (T1, T31, and T32; see Table 1 and Fig. 2).

The D-dimer levels at the first presentation (T-60) were significantly higher in dogs with severer clinical signs (*P* < 0.05; see Table 1 and Fig. 2). Pathological level of D-dimer was found in 100% of class III dogs only. However, no dog in any group had higher than the reference interval of D-dimer level at the end of the study (T182). The D-dimer levels in all classes of the dogs decreased significantly after initiation of the therapy (*P* < 0.05; see Table 1 and Fig. 2). The D-dimer levels did not change significantly after melarsomine injection (T1, T31, and T32; see Table 1 and Fig. 2).

The CRP levels at T-60 were significantly higher in dogs with severer clinical signs (*P* < 0.05; see Table 1 and Fig. 2). Pathological level of CRP at T-60 was found in 50% in class II and 100% in class III groups. The CRP levels were persistently higher during the therapeutic period. In class II and class III, 25% and 100% of dogs, respectively, had pathological levels of CRP at T182. Although the CRP levels in all classes of dogs decreased significantly after initiation of the therapy (*P* < 0.05; see Table 1 and Fig. 2), CRP levels increased steeply after melarsomine injection (see data on T7, T30, T31, T32 and T39; see Table 1 and Fig. 2).

The IL-6 levels at T-60 were significantly higher in dogs with severer clinical signs (*P* < 0.05; see Table 1 and Fig. 2). Pathological level of IL-6 at T-60 was found in 50% of class II and 100% of class III groups. The IL-6 levels were persistently higher during the therapeutic period, although only 50% of the class III dogs had pathological levels of IL-6 at T182. Although the IL-6 levels in all classes of dogs decreased significantly after initiation of the therapy (*P* < 0.05; see Table 1 and Fig. 2), IL-6 levels increased steeply after melarsomine injection (see data on T7, T30, T31, T32 and T39; see Table 1).

## Discussion

Although several therapeutic protocols have been proposed [2–7], adverse reactions related to adulticide therapy are inevitable; and, thus, close monitoring of the patient status during the therapeutic period is critical for successful and safe treatment of dogs with HWD.

Several recent studies evaluated the role of biomarkers in assessing the severity of HWD in dogs [4, 13–15, 20, 21]. Also, several cardiopulmonary, inflammatory and renal markers have been studied and found to be beneficial for practitioners to assess undetectable cardiopulmonary damages clinically during adulticide therapy [4, 13–15, 20, 21]. These studies found that dogs with severer clinical signs had higher levels of biomarkers and, thus, might have higher risk of adverse effects during adulticide therapy. In general, the severity of complications related to HWD treatment is closely associated with worm burden, host reaction to the parasite, the duration of the infection and the amount of exercise during therapeutic period [21, 22]. Our study also suggested that the severity of complications assessed by serum biomarker levels might be higher in dogs in more advanced classes of HWD.

cTnI is a cardiac biomarker for detecting myocardial injuries in dogs [12] Several studies found elevation of serum cTnI levels in dogs with HWD [13, 14] A recent study found that serum cTnI levels in dogs in classes I and II were not significantly different from those of healthy control dogs [20] Pathological elevation of serum cTnI levels was observed only in dogs in advanced classes of HWD (III and IV) [20]. Similar to this study, our study also found higher cTnI levels in dogs with class III HWD. All dogs in class III had higher cTnI levels than the reference interval. Similar to the results of the previous study [20], serum cTnI levels in class I were not found to be significantly elevated in this study. However, unlike the previous study [20], all dogs in class II had higher serum cTnI levels than the reference interval, although the mean cTnI levels were much lower than those in class III. This difference implied that our study population might have suffered HWD for a longer period than the dogs in the other study and, thus, had higher levels of cTnI in dogs with mild clinical signs (class I) because cardiac damage from HWD is produced chronically.

A decrease in cTnI levels after adulticide treatment has been reported [15]. In this study, the serum cTnI levels reduced significantly after adulticide therapy. Interestingly, serum cTnI levels in class III decreased significantly after doxycycline and ivermectin premedication, although the levels in classes I and II did not differ significantly. This result suggested that pretreatment with macrocyclic lactone and doxycycline before adulticide therapy can potentially reduce the myocardial damage at least in dogs with severe clinical signs. The exact mechanism was not clear; however, pretreatment against microfilaria and *Wolbachia* spp. might be involved in reducing myocardial damage from HWD. Most complications related to adulticide therapy occurred after melarsomine injection. However, this study clearly demonstrated that the level of cTnI was not affected by melarsomine injection, because the serum cTnI levels decreased consistently during the therapeutic period regardless of the administration of melarsomine injection. This study also demonstrated that myocardial damage can be alleviated by reducing the number of worms. Therefore, complications related to adulticide therapy might be more closely related not to cardiac damage but to the intense pulmonary inflammatory process.

D-dimer is a by-product of fibrinogen degradation and is an evidence of hypercoagulability in dogs with various thromboembolic diseases, including parvoviral enteritis, immune-mediated hemolytic anemia and HWD [14–17, 23]. Carretón et al. [20] have found that the concentrations of D-dimer in dogs in classes I and II were within the normal reference interval, while those in dogs in classes III and IV were much higher than the reference interval, indicating the occurrence of significant thromboembolic processes in dogs with an advanced stage of HWD. Similar to the previous study, this study also found that the serum D-dimer levels were markedly increased only in dogs in class III of HWD. Although the previous study found that only 40% of the class III dogs had pathological levels of D-dimer, this study found that 100% of the dogs in class III had pathological levels of D-dimer at the first presentation of the study. In this study, a higher percentage of dogs in class III having pathological levels of D-dimer might be because our study population was affected more by the contributing factors (ie, host immune reaction to the clearance of dead worms, duration of infection and ability to restrict exercise) involved in the complications of HWD in dogs.

In general, complications of pulmonary thromboembolism related to adulticide therapy occur from the death of the parasites [24]. Therefore, the authors expected to see elevation of D-dimer levels after melarsomine injection. In this study, D-dimer levels increased after the second and third melarsomine injections and reduced significantly a week after each melarsomine injection. The elevation in D-dimer levels seemed to be higher and maintained for a longer duration after melarsomine injection in dogs with severer clinical signs. However, compared to other another study [21], the level of elevation in D-dimer was less severe in this study. Carretón et al. [21] suggested that steroid therapy during adulticide therapy might contribute to the elevation of D-dimer, as also noted in other studies [22, 25]. One study also found that the levels of elevation of D-dimer were different from the therapeutic protocol, and the classic 2-dose melarsomine injection protocol could induce the highest mean D-dimer concentrations in dogs with HWD [21]. Since this study did not use steroids during adulticide therapy in order to minimize their influence on serum CRP and IL-6 levels, the lower elevation level of D-dimer after melarsomine injection might be attributed to the absence of steroid supplementation. This study suggested that the risk of pulmonary thromboembolism might be greater after melarsomine injection, and dogs with severer clinical signs had a greater risk of pulmonary thromboembolism during adulticide therapy, as reported previously [25, 26]. Furthermore, as suggested by Carretón et al. [21], routine use of steroids in dogs with HWD should be reconsidered. To justify the use of steroid therapy in dogs with HWD, clinical trials for assessing the benefits and drawbacks of steroid therapy should be conducted before the HWD therapeutic guidelines recommend the routine use of steroids.

CRP is a hepatic-origin acute-phase protein and increases following IL-6 secretion by macrophages and T cells in response to inflammation [27]. A recent study found close correlation between CRP and IL-6 in patients with high risk of cardiovascular disease [28]. During infection with heartworm disease, various degrees of inflammation occur as result of vascular and pulmonary tissue damage caused by adult worms, microfilariae, and *Wolbachia* spp. [24]. Although serum level of IL-6 has never been studied in dogs with HWD, a recent study found that CRP levels were significantly higher in dogs with HWD and were closely correlated to the severity of pulmonary hypertension [4]. Furthermore, another study also found that the CRP levels increased with the severity of the disease [20]. Similar to the previous studies, the CRP levels were higher in dogs in classes II and III in this study. Dogs with severer clinical signs had higher CRP and IL-6 levels at the first presentation. Interestingly, doxycycline therapy against *Wolbachia* spp*.* during premedication could have dramatically reduced the levels of CRP and IL-6 in this study, suggesting that the role of *Wolbachia* spp*.* in the inflammatory process and pathogenesis of HWD is substantial, as noted by another study [29].

In addition, this study interestingly found that melarsomine injection could lead to proinflammatory response during adulticide therapy. The CRP and IL-6 levels increased after each melarsomine injection and were the highest seven days after each melarsomine injection. Furthermore, the levels of elevation in CRP and IL-6 were higher in dogs with severer clinical signs after melarsomine injection. The trend of elevation in CRP and IL-6 during adulticide therapy suggested that both the markers might be closely related, as noted in a human study [28]. Unlike CRP, IL-6 in dogs in class II was maintained at higher levels for a longer period in this study, suggesting that IL-6 might be more sensitive for reflecting the inflammatory process in dogs with milder clinical signs. The cytokines were found to have persistently higher levels 2–3 months after melarsomine injection, suggesting that a longer therapeutic period against the inflammatory process might be required in dogs. Unlike dogs in classes II and III, dogs in class I had no pathological levels of CRP and IL-6, except for a short period after melarsomine injection, suggesting that no anti-inflammatory therapy is necessary for asymptomatic dogs with HWD.

The findings on CRP and IL-6 concerned us about the use of steroid therapy in dogs with HWD. Our cytokine analyses clearly proved that potent anti-inflammatory therapy during adulticide was necessary (ie, probably for a longer period than the AHS guidelines indicate, especially in dogs with severe clinical signs), while steroid therapy could increase the risk of pulmonary thromboembolism in dogs with HWD, as noted in the D-dimer analysis. Therefore, the use of steroids should be decided by carefully weighing the benefits and drawbacks of this therapy. A large-scale clinical trial is required to prove the benefits of steroids in treating HWD in dogs. Furthermore, this study suggests that minimal doses of steroids for alleviating clinical signs should be maintained; and prophylactic use of steroids in asymptomatic cases may not be necessary.

Significance of the study results was limited by the small sample size; and thus, further studies with a large sample size should be conducted to verify our results. Furthermore, the steroid was not given to any dogs to minimize its influence on cytokine levels in the study population, although the authors tried to adhere strictly to the 2014 AHS guidelines. Therefore, the trend of cytokine levels might be different in dogs when the 2014 AHS guidelines are strictly followed. Despite these limitations, this study suggested that the levels of serum biomarkers related to cardiac injury, thromboembolism and inflammation increased as the severity of HWD worsened, as reported in other studies [4, 13–15, 20, 21]. Furthermore, dogs with severer clinical signs might have a greater risk of post-adulticide complications. Therefore, a proper decision on the clinical status of HWD in each affected dog based on meticulous diagnostic testing, including biomarker analysis, should precede the initiation of adulticide therapy.

## Conclusion

This study found that monitoring of inflammatory and hemostatic markers in dogs with heartworm disease being treated with melarsomine might be beneficial in predicting the clinical outcomes and complications associated with melarsomine treatment.
